# Association of participants who screened positive for night eating syndrome with physical health, sleep problems, and weight status in an Australian adult population

**DOI:** 10.1007/s40519-023-01603-x

**Published:** 2023-09-20

**Authors:** Sai Janani Sakthivel, Phillipa Hay, Stephen Touyz, David Currow, Haider Mannan

**Affiliations:** 1 Translational Health Research Institute, Western Sydney University, Campbelltown, NSW 2560 Australia; 2 Mental Health Services, SWSLHD, Camden and Campbelltown Hospitals, Sydney, Australia; 3 Inside Out Institute, University of Sydney, Sydney, Australia; 4 Sydney Local Health District, Sydney, Australia; 5 University of Wollongong, Wollongong, NSW Australia; 6 ImPACCT, Faculty of Health, University of Technology Sydney, Sydney, Australia; 7 Repatriation General Hospital, Flinders University, Adelaide, Australia; 8 Wolfson Palliative Care Research Centre, University of Hull, Hull, UK

**Keywords:** Night eating syndrome, Physical health, Sleep problems, Weight status

## Abstract

**Background:**

Night eating syndrome (NES) is a unique eating disorder characterised by evening hyperphagia and nocturnal ingestions which cause significant distress and/or impairment in functioning. Despite the growing literature, NES remains poorly understood and under diagnosed. As such, this study aims to compare the prevalence of physical health conditions in participants with NES when compared to participants without an eating disorder (ED) and participants with other eating disorders (including anorexia nervosa (AN), binge eating disorder (BED) and bulimia nervosa (BN)) in a general population Australian sample of adults.

**Methods:**

The data for this study were obtained from the 2017 Health Omnibus Survey (HOS) a multi-stage, cross-sectional survey, conducted by Harrison Research in South Australia. This current study focused on 2547 participants over 18 years of age and specific questions from this population survey including those related to participant demographics and health.

**Results:**

This study identified that participants who screened positive for night eating syndrome (spNES) when compared to participants with other eating disorders (ED) or no ED diagnosis, were significantly more likely to have an increased age, be female, have lower levels of education and have lower household income. Additionally, the spNES group was significantly associated with sleep apnoea (*p* = 0.031), insomnia or other sleep problems (*p* < 0.0001), increased BMI (*p* < 0.0001), increased levels of pain/discomfort and lower physical health-related quality of life. Hypertension, hypercholesterolemia, and diabetes were not significantly associated with the spNES group or the “other ED” group which included participants with AN, BED, BN.

**Conclusions:**

Several physical health problems were found to be significantly associated with the spNES group including sleep problems, increased BMI, increased levels of pain and lower self-reported physical health-related quality of life. Consequently, future research exploring the complex interaction between NES and these medical conditions may provide further insight into the diagnosis, screening tools and management of NES. Additionally, this study highlights the need for future studies which use larger population-based samples.

**Level of evidence:**

Level III. Evidence obtained from well-designed cohort or case–control analytic studies.

## Introduction

Eating disorders (ED) are physically, physiologically, financially, and socially incapacitating, contributing to a significant burden for individuals and society [1, 2]. ED affects ~ 4% of the Australian population, however, the actual prevalence may be significantly higher [2]. These disorders include anorexia nervosa (AN), binge eating disorder (BED), bulimia nervosa (BN), other specified and unspecified feeding and eating disorders (OS/UFED. Night eating syndrome (NES) is classified as a type of OSFED [3].

NES is characterised by recurrent episodes of night eating (NE), described by excessive food consumption after the evening meal (evening hyperphagia) or eating after awakening from sleep (nocturnal ingestions) [3, 4]. Individuals with NES are aware and can recall these episodes [3, 5]. Additionally, Allison et al. proposed that this pattern of disordered eating must be maintained for 3 months or more and with specific features such as at least 25% of food intake consumed after the evening meal and at least 2 episodes of nocturnal eating per week [5]. Although the aetiology of NES is poorly understood, the syndrome is thought to result from a desynchronisation of mood, sleep, satiety, and circadian rhythms of food ingestion [4, 6, 7]. Additionally, NES has a significant association with concurrent psychiatric diagnoses and comorbidities including BED, BN, generalised anxiety disorder and substance use disorder [4, 8]. Consequently, individuals with NES often experience significant distress and/or impairments in normal functioning [3, 4, 6]. With a prevalence of 1.5% in the general population of the United States [9], NES creates a substantial burden to the healthcare system, detrimentally impacting quality of life and increasing morbidity [4, 6, 8, 10, 11].

Currently, inconsistent evidence exists regarding the impact of NES on body mass index (BMI; kg/m^2^), physical health and sleep [4, 6–8, 11–13]. Additionally, a majority of the research on NES has been conducted within clinical settings [14–16] or in adolescent populations [17–19]. As such, there is a lack of knowledge regarding NES within general and adult population groups [14, 20, 21]. A study, reported in 2012, conducted in an adult Swedish twin population by Lundgren et al. reported no association between a lifetime history of any physical health variable (specifically blood pressure, cholesterol, gastrointestinal problems, heart burn and diabetes) and evening hyperphagia as well as nocturnal ingestions of food [22]. It is likely that the inconsistencies have occurred due to the use of different samples and age groups and highlights the importance of replication of studies. In contrast, this study primarily aims to investigate the prevalence of physical health conditions (specifically; hypertension, hypercholesterolemia, diabetes/ high BSL, and sleep apnoea), sleep problems, low physical health-related quality of life [23] and increased weight status [24] in people who screened positive for night eating syndrome (spNES) compared to people without an eating disorder and people with BED or BN or AN in a general population Australian sample of adults.

## Methods

### Study design and weighting

The data for this study were obtained from the 2017 Health Omnibus Survey (HOS) [25, 26], a multi-stage, cross-sectional, face-to-face survey, conducted by Harrison Research in association with South Australia Health. This 25-page structured interview was conducted annually and included questions related to demographics and participant health (medical conditions, weight control, etc.). Between September and December 2017, personal interviews were conducted with households throughout South Australia. Additionally, cities and towns were initially stratified into Metropolitan and Country statistical areas based on the Australian Bureau of Statistics (ABS) census. From the Statistical Areas Level 1 (SA1) used in the ABS 2016 census, 398 metropolitan and 132 regionals were selected through a probability of selection proportional to their size procedure. Furthermore, a skip pattern of every fourth household, was utilised to select ten households within each level 1 SA and only one interview with a participant 15 or over was conducted per household. In instances where more than one person aged 15 years or over resided within the household, the respondent was the participant with the most recent birthday. Verbal consent was obtained for adult participants and written consent was obtained from parents or guardians of participants under the age of 18 years. Additionally, missing data were obtained by telephone interview and weighted by ABS 2016 Census data on age, sex, educational level, country of birth, marital status and household income.

### Participants

From the 5300 households selected in 2017, 2977 interviews were conducted between September and December 2017, with a response rate (i.e. included vacant houses, holiday homes, businesses and non-permanent tenants) of 57.0% and a participation rate (i.e. the number of people eligible to participate excluding those not able to be contacted after six attempts (i.e. house was vacant at the time of survey) was 65.3% [25, 26]. This study reports only data collected in adult participants (i.e. data from all participants under 18 years of age were excluded).

### Ethics

The 2017 HOS [25, 26] was approved by the University of Adelaide Human Research Ethics Committee (HREC). Ethics Approval ID: H-097-2010. An exemption from further Human Research Ethics Review was obtained from the Western Sydney University HREC as data may be analysed and published beyond 2019 on the condition that the datasets are fully de-identified prior to being shared with researchers that were not included on the original ethics approval and analysis aligns with consent provided by participants.

### Measures

#### Demographics

Sociodemographic data were collected by face-to-face interview. During the initial interview, participants self-reported their height and weight, and BMI (kg/m^2^) was calculated and classified in accordance with the Centers for Disease Control and Prevention (CDC) [24].

#### Eating disorder symptoms

Questions on eating disorder symptoms were derived from the Eating Disorder Examination Interview (EDE) [27], to ensure participants were accurately allocated into the BN, BED and spNES groups. Participants were also interviewed with questions clarifying components of DSM-5 [3]. Participants were asked to respond based on the 3 months immediately preceding the interview, to screen for NE, BN, BED, AN or no eating disorders. These questions included episodes of night eating, episodes of binge eating, purging, the presence of distress and other emotions associated with these episodes, dietary restriction, presence of inappropriate compensatory behaviours and level of weight/shape over-valuation. Frequency of binge eating, and night eating episodes were characterised by, “Not at all”, “less than weekly”, “once a week”, “two or more times a week”, “don’t know” or “refused”. The presence of inappropriate compensatory measures (e.g., laxatives, diuretics, etc.) was categorised by “yes”, “no” and “refused”. The question to determine night eating was: “*In the past 3 months have you had any episodes of night eating? By night eating I mean waking from sleep and eating (i.e. you were not sleep walking and eating, you were awake), OR episodes of eating a very large amount after your evening meal. (This does not include eating at night because of social or other circumstances e.g., you are travelling overseas on a night flight or because of work shifts)*”. Participants responded to questions regarding distress associated with binge eating and night eating with the following responses: “not at all”, “yes—a little”, or “yes—a lot” or with “refused”. For this study, the spNES variable was defined by participants with any episodes of night eating with “a lot” of distress experienced over the last 3 months.

#### Physical health questions

Participants self-reported a medical diagnosis of hypertension, hypercholesterolemia, high blood sugar level (BSL) or diabetes, insomnia, and obstructive sleep apnoea, with the question, “Has a doctor ever told you that you have …”. These questions were answered with “yes”, “no”, “don’t know” or “refused”. Additionally, participants could answer, “No, not told have any of these conditions”. Participants who responded with “don’t know” or “refused” were reported as “missing”. Furthermore, pain and discomfort were rated on a scale from 1 to 5, described as “no”, “slight”, “moderate”, “severe” and “extreme”.

#### Physical health-related quality of life (HRQoL)

Participant health status was assessed with the validated Short Form 12 Health-Related Quality of Life (SF12) tool [28]. The SF-12 [28] is a 12-item questionnaire which contains 8 subscales: physical functioning, role limitation due to physical problems, bodily pain, general health perceptions, vitality, social functioning, role limitations due to emotional problems and mental health. These subscales are weighted in 2 separate scales, a Physical Component Summary Scale (PCS) and a Mental Component Summary Scale (MCS) [28]. Each scale has a mean score of 50 and standard deviation of 10, with higher scores indicating an increased health status. This current study utilised only the PCS to identify the impact of physical health problems on quality of life [28].

### Data analysis

The data were inspected for normal distribution and cleaned. Participants below the age of 18 years were removed. All data analyses were performed using “Statistical Packages For Social Sciences” (SPSS Version 27) and “Statistical Analysis System” (SAS version 9.4). Adjusted prevalence estimates and their respective 95% confidence intervals (CI) were predicted and reported as percentages. For this study, the spNES variable was defined as participants experiencing “a lot” of distress associated with any episodes of night eating which had occurred for the last 3 months. Additionally, participants were divided into the “other ED group” if they currently satisfied DSM-5 diagnostic criteria [3] for AN, BED or BN using the EDE-Q [27]. The “no known ED group” consisted of the remaining participants above 18 years of age.

Descriptive data were expressed in percentage of total participants who responded to the question, means, medians, standard deviations, and interquartile ranges. Chi-squared (χ^2^) test, Kruskal–Wallis test and *F* test were used as appropriate, to assess differences between the 3 groups and their associations with demographic and physical features. Physical characteristics such as BMI [24], physical component of the SF12 [28] [utilised to represent physical health-related quality of life (HRQoL)] and medical conditions (including increased BMI, hypercholesterolemia, hypertension, hyperglycaemia or diabetes mellitus, sleep apnoea, insomnia and other problems) were examined whilst controlling for confounding variables (i.e. age and sex). Regression analyses which adjusted for age and sex were utilised to examine the relationship between the dependent outcome (physical features) and the independent variables (spNES, other ED or no ED). Linear regression was used for continuous data whilst ordinal logistic regression was used for ordinal data.

### Outcomes

There were 23 people (0.90%) with spNES, 44 (1.73%) with other EDs and the remaining 2480 (97.37%) with no ED. Table 1 presents this and the association between the spNES, other ED and no ED groups and socio-demographic features including age, sex, marital status, education, and household income. The spNES variable was significantly associated with the female sex, increased age, reduced highest education level and lower household income. Additionally, the “other ED” group had increased participants in the certificate, trade or apprenticeship groups when compared to the “no ED” group.

**Table 1:** Demographic and clinical features of study participants over the age of 18 years

Characteristic (*n*)	Total *n* = 2547	spNES *n* = 23	BN, AN, BED (other ED) *n* = 44	No ED known *n* = 2480	Statistics	Post Hoc
	Mean (SE) *n*				ANOVA (*p*)	
Age (2547)	49.02 (0.36) 2547	45.32 (2.70) 23	38.18 (1.97) 44	49.25 (0.38) 2480	*F* = 8.04 *df* = 2 (*p* < 0.0001)	No ED > NE > Other ED

Characteristic (*n*)	*N* (%)				χ^2^ (*p*)	Post Hoc
Sex (2548)						
Female	1307 (51.30)	18 (78.26)	31 (70.45)	1258 (50.72)	χ^2^ = 13.505 *df* = 2 (*p* = 0.001)	NE and Other ED more likely to be female than no ED
Male	1241 (48.70)	5 (21.74)	13 (29.55)	1223 (49.31)		
Marital status (2543)						
Married/De facto	1617 (63.59)	10 (43.48)	24 (54.54)	1583 (63.83)	χ^2^ = 8.758 *df* = 4 (*p* = 0.067)	N/A
Never married	540 (21.23)	7 (30.43)	15 (34.09)	518 (20.89)		
Separated/divorced/widowed	386 (15.18)	6 (26.09)	5 (11.36)	375 (15.12)		
Highest Education level (2544)						
Secondary school	921 (36.20)	11(47.83)	11 (25.00)	899 (36.25)	χ^2^ = 10.228 *df* = 4 (*p* = 0.037)	Other ED more certificate and trade and apprenticeship than no ED
Certificate/trade/Apprenticeship	962 (37.81)	8 (34.78)	26 (59.09)	928 (37.42)		
Bachelor or higher	661 (25.98)	4 (17.39)	7 (15.91)	650 (26.21)		
Household Income (2006)						
Up to $40,000	574 (28.61)	11(47.83)	11 (25.00)	552 (22.26)	χ^2^ = 13.386 *df* = 6 (*p* = 0.037)	NE lower income (< 40 K) than no ED
$40,001–$100,000	775 (38.63)	8 (34.78)	21 (47.73)	746 (30.08)		
$100,001–$140,000	307 (15.30)	1 (4.35)	7 (15.91)	299 (12.06)		
$140,001 or more	350 (17.45)	1 (4.35)	2 (4.55)	347 (13.99)		

Four cases of BN also have NE and were included with the “Other ED” group

“spNES” group = participants with night eating and experienced distress, “Other ED” group = participants with Anorexia nervosa (AN), Binge eating disorder (BED) and Bulimia nervosa (BN), “No ED” group = participants who are not categorised into the “NE group” or “Other ED” groups

*F* is calculated by dividing two mean squares, *Df is* degrees of freedom

Table 2 reports the associations between self-reported physical health conditions and spNES, other ED and no ED groups. Sleep apnoea was significantly associated with the spNES variable (*p* = 0.031). Similarly, insomnia or other sleep problems and the spNES group had a significant relationship (*p* < 0.0001). The mean BMI for participants who spNES was 33.45 kg/m^2^, which is higher than participants in the other ED group and significantly higher than participants without a known ED. Hypertension, hypercholesterolemia and diabetes were not significantly associated with an ED diagnosis. However, near significance was reached when exploring the association between diabetes, high BSL and in participant who spNES.

**Table 2:** Comparison of physical health problems between people with NES and other groups

Characteristic	spNES *n* = 23 (0.90%)	BN, AN, BED (other ED) *n* = 44 (1.73%)	No ED known *n* = 2480 (97.33%)	Statistic	Post hoc
Characteristic (*n*)	*N* (%)			χ^2^ (*p*)	
Hypertension (1807)	12 (52.17)	10 (22.73)	687 (27.70)	χ^2^ = 4.285 *df* = 2 (*p* = 0.117)	N/A
Hypercholesterolemia (1807)	7 (30.43)	14 (31.82)	563 (22.70)	χ^2^ = 0.397 *df* = 2 (*p* = 0.820)	N/A
Diabetes or high BSL (1808)	8 (34.78)	7 (15.91)	278 (11.21)	χ^2^ = 5.425 *df* = 2 (*p* = 0.066)	N/A

Characteristic (*n*)	*N* (%)			Binary logistic regression (*p*)	Post hoc
Sleep apnoea (1808)	5 (21.74)	3 (6.82)	123 (4.96)	OR = 0.711 SE = 0.159 B = -0.342 (*p* = 0.031)	NE > other ED > no ED
Insomnia or other sleep problems (1807)	11 (47.83)	10 (22.73)	219 (8.83)	OR = 0.646 SE = 0.108 B = -0.437 (*p* < 0.0001)	NE > other ED > no ED

Characteristic (*n*)	Median (IQR) *n*			Statistic K-W	Post hoc
Pain/discomfort (2545)	2 (2–4) 23	1 (1–2) 44	1 (1–2) 2477	*c* index = 0.628	NE > other ED > No ED

Characteristic (*n*)	Mean (SD) *n*			ANOVA (*p*)	Post hoc
BMI (kg/m^2^) (2403)	33.45 (9.41) 22	31.37 (7.90) 44	26.75 (5.55) 2337	*F* = 0.215 *df* = 3 *B* = 0.197 95%CI = 2.678 (*p* < 0.0001)	NE > other ED > No ED
Physical component (SF12) (2533)	42.90 (12.98) 22	45.94 (10.34) 44	48.07 (10.60) 2467	*F* = 3.55 *df* = 3 *B* = − 0.82 95%CI = − 1.006 (*p* < 0.0001)	NE < other ED < No ED

Four cases of BN also have NE and were included with the “other ED” group

“spNES” group = participants with night eating and experienced distress, “other ED” group = participants with anorexia nervosa (AN), binge eating disorder (BED) and bulimia nervosa (BN), “No ED” group: participants who are not categorised into the “NE group” or “other ED” groups

*F is* calculated by dividing two mean squares, *Df* is degrees of freedom

Higher levels of self-reported pain and discomfort were significantly associated with the spNES variable, with an interquartile range of 2–4. In comparison, participants in the other ED and no ED groups had an interquartile range of 1–2. Participants who spNES were found to have 3.21 times higher odds of low pain compared with no pain, 2.39 times higher odds of moderate pain compared with no pain and 6.16 times higher odds of extreme pain compared with no pain. As such, the odds of the spNES group were highest when participants reported extreme pain. These finding were all statistically significant. When compared with no pain, participants in the “other ED” group were found to have 1.16 times higher odds of low pain, 1.91 times higher odds of moderate pain and 2.46 times higher odds of extreme pain. Thus, for participants with AN, BED or BN, the odds of the self-reported pain increased with the severity of pain reported. The findings for the “other ED” group were all statistically significant except for when participants reported “low pain”.

This study also identified a significant association between participants who spNES and poorer scores for the summary physical component of the SF-12 [27]. The Physical Component Summary Scale (PCS) [27] involves several questions categorised into limitations to physical functioning, limitations to usual role activities, presence of bodily pain and general health perceptions. This suggested that participants in the “spNES” group had statistically significantly lower HRQoL than participants within the “No ED” group.

## Discussion

This study investigated the complex interaction between participants who spNES and physical health features within a representative adult Australian population, to provide insight regarding the pathophysiology and thus, the management of NES and other ED. The respondents were contacted independently of health service contact in distinction to previous studies. This current study found that participants who spNES were more likely to be female, less likely to have obtained higher education levels and had lower household income when compared to those with no ED. This reiterates the findings of a previous epidemiological study conducted in 2014, which speculated this higher prevalence of NES in women was attributable to the lack of distress reported by men, although more men endorsed night eating symptoms [29]. Additionally, this study identified that those who spNES much like other EDs such as BED had notable physical health comorbidities [30, 31].

Sleep apnoea and insomnia or other sleep problems were identified to have a significant relationship with participants who spNES. This echoed current literature [32–36] which revealed significant relationships between poor sleep quality and NES when utilising self-reported questionnaires. This contrasted with Cleator et al. [32] and Geliebter et al. [16] who reported no significant association between OSA and NES. Although objective data were obtained using polysomnography which objectively evaluated the Apnoea–Hypopnoea Index (AHI), Geliebter et al. [16] were limited in providing further information addressed through questionnaires such as sleep duration, daytime sleepiness, and impact of quality of life. Confirming the current literature, a narrative review [37] identified an association with nocturnal feeding and impaired sleep when observing shift workers, suggesting nocturnal feeding mimicked NES and that this disturbance in circadian rhythm resulted in metabolic disorders.

Although limited studies have been written on this specific topic, Karp et al. [38] suggests that this may be associated with increased BMI which is often linked with NES. Furthermore, literature suggests that repeated ingestion of large amounts of food may be associated with gastric distension and subsequently pain [39, 40]. A more speculative explanation is that people who have chronic pain may experience night awakenings from such pain and begin a pattern of overeating when aroused from sleep with pain [39, 40]. This is consistent with Beauchamp et al. who suggested pain was associated with poor sleep quality, which was more central to the NES symptom network than nocturnal ingestions and evening hyperphagia when using a network analysis of the diagnostic criteria for NES [41].

Literature concerning the relationship between NES and BMI provided inconclusive and contradictory findings [16, 32, 36, 42, 43]. This current study identified a significantly positive relationship between participants who spNES and BMI when using self-reported height and weight. Several studies hypothesised that the incongruent results when exploring the association between NES and BMI may result from the use of different measurement methods and the presence of confounding variables such as age, sex, socio-economic status, and symptoms variability [43]. The present sample controls for such confounding factors as selection bias is lower in representative community populations, lending more confidence to the finding of an association with BMI.

This study identified that the spNES group was significantly associated with lower scores on the physical component of the SF12 [28] (i.e. physical health-related quality of life (HRQoL). This questionnaire explored several facets of physical health including patient reported limitations to physical functioning, limitations to usual role activities. Current literature [17, 36, 42] exploring the relationship between NES and physical activity reported incongruous findings. It was suggested that this may result from the use of different tools when assessing activity or use of small sample sizes. Several studies have suggested multifactorial causes for participant reported poorer health-related quality of life, including sleep quality, age and stress levels [17, 44]. This was particularly evident when observing university participants [17, 44]. The poorer sleep quality reported in participants with NES, as well as the presence of other physical health problems such as pain, may therefore explain the poorer physical HRQoL we found.

The current data suggested that the spNES variable was not significantly associated with diabetes mellitus or hyperglycaemia, hypercholesterolemia, or hypertension. This supported existing studies [32, 33, 35] which reported no significant relation between NES and type two diabetes mellitus, as well as other concomitant disease including hypertension, hypercholesterolemia, and cardiovascular disease. Hood et al. however, reported a significant correlation between NEQ scores and haemoglobin A1c (HbA1C) [34], an indicator of glycaemic control. Current literature [32–35] regarding the impact of NES on these conditions have largely been conducted within clinical environments, as such these studies may not be representative of the general population, with participants more likely to have comorbidities [32, 34, 35].

This study has several clinical implications. NES is an emerging area for clinical investigation, evaluation, and intervention, due to its substantial impact on individuals and the healthcare system [3, 4, 6, 8, 10, 11]. The findings of this study highlight the need for health professionals to be aware of the extensive impacts of NES and other eating disorders on patient health and their associations with comorbidities. Screening for specific comorbidities such as sleep apnoea will allow for earlier diagnosis and management of these conditions, thus improving patient reported quality of life.

### Strengths and limitations

The study has several strengths and limitations. It reported on participants who were representative of the general population, with males and females evenly distributed, and with a wide range of ages. The validity of this current study was increased through the use of a representative sample, which reduced the likelihood of bias and confounding variables which may occur from selection bias and bias in clinical samples. Additionally, the use of face-to-face interviews enabled participants to clarify any survey questions to obtain the most accurate data. Similarly, categorisation into spNES, other ED and no ED groups was achieved through interview items which closely matched the DSM-5 [3] and ICD-11 [45] criteria, thus increasing the validity of this study. However, the proposed diagnostic criteria suggested by Allison et al. is often used in current literature [5], which categorises NES into evening hyperphagia and nocturnal ingestions. This study would be improved with use of food records over a period to assess evening hyperphagia and the inclusion of questions regarding the frequency of nocturnal ingestions. Additionally, due to the variety of criteria utilised in current literature to diagnose NES [11], this study may have included participants in the spNES group who may not meet the “full diagnostic criteria”.

The data set relied heavily upon self-reported data to lay (albeit trained) interviewers, which may have over-estimated number of participants with each medical condition. Furthermore, this study did not investigate the cases of self-reported diabetes or high BSL to identify type of diabetes or severity. Potential bias of self-reported data could not be excluded; however, the validity of self-reported data for these medical conditions has been proven by several studies [46, 47] and is commonly utilised in screening and surveillance programmes [48, 49]. Participant height and weight were self-reported to calculate BMI, so clinician-measured anthropometric may have yielded more accurate results [50]. However, the direction and magnitude of findings have face validity, and most people are likely to report with the same degree of skewness—i.e. under-reporting. The use of estimated anthropometric measures in place of accurate measures reduced the accuracy of results and consequently, the validity of diagnoses.

### What is already known on this subject?

Existing literature regarding the association of NES on components of physical health is inconsistent and has been conducted largely in clinical settings [14–16] or within adolescent populations [17–19]. As such, further research is imperative to understand the impact of NES within general representative and adult population samples [14, 20, 21].

### What this study adds?

This current study aims to investigate the prevalence of physical health problems in spNES when compared to individuals without an eating disorder or those with other eating disorders (BED or BN or AN) in a general population Australian sample of adults.

## Conclusion

NES is an emerging area for clinical investigation, evaluation, and intervention, due to its substantial impact on individuals and the healthcare system [3, 4, 6, 8, 11]. This study identified significant associations between the spNES variable and multiple facets of physical health including higher BMI, increased levels of pain/ discomfort, poorer scores of the physical component of the SF-12, sleep apnoea, insomnia or other sleep problems. The spNES group was not found to be significantly associated with diabetes mellitus or hyperglycaemia, hypercholesterolemia, or hypertension in this study. Future longitudinal studies on general population samples are essential to clarify the impact of NES on physical and mental health to improve the assessment, identification of comorbidities and management of NES.

## Data Availability

Data is not publicly available.
